# Differential resistance to nematode infection is associated with the genotype- and age-dependent pace of intestinal T cell homing

**DOI:** 10.1038/s41598-024-76204-4

**Published:** 2025-02-05

**Authors:** Joshua Adjah, Bhavya Kapse, Hongwei Zhang, Susanne Hartmann, Sebastian Rausch

**Affiliations:** Department of Veterinary Medicine, Institute of Immunology, Freie Universität Berlin, 14163 Berlin, Germany

**Keywords:** Immunology, Mucosal immunology

## Abstract

The resistance of inbred mice to nematode infections varies depending on the extent of protective Th2 responses. Here, we compared two mouse lines differing in resistance to infection with the enteric nematode *Heligmosomoides polygyrus bakeri* despite the similar instruction of GATA-3+ T effector cells. Resistant BALB/c mice rapidly recruited high numbers of Th2 cells to the gut within the 1-week time frame required for larval development in the intestinal submucosa. C57BL/6 mice failed in the optimal control of early nematode fitness, with mucosal Th2 response peaking after 2 weeks when the larvae had left the tissue and relocated to the gut lumen as adult worms. The faster homing of Th2 cells to the gut of BALB/c mice is related to the extensive expression of the chemokine receptor CCR9 in GATA-3+ cells and higher frequencies of aldehyde dehydrogenase expressing dendritic cells present in mesenteric lymph nodes. Furthermore, nematode-infected older BALB/c mice displayed impaired resistance due to delayed mucosal homing of effector cells, which synergized with more numerous Th2/1 hybrid cells acting as IFN-γ-dependent confounders of type 2 responses. Hence, the distinct kinetics of effector cell recruitment to the infected gut and the quality of GATA-3+ T cell responses contribute to the genotype- and age-dependent resistance to intestinal nematode infections.

## Introduction

Nematodes infecting the gastrointestinal tract are among the most common parasites leading to extensive morbidity in humans, livestock, and companion animals worldwide^1,2^. *Ascaris*, whipworms, and hookworms account for the majority of human gastrointestinal (GI) nematode infections. Due to the long persistence of the adult worms and the slow development of protective immunity, *Ascaris* and whipworm burdens accumulate during childhood and reach a peak shortly before adolescence, followed by recurrent low-intensity infections throughout adulthood^1,3–5^. For unclear reasons, both the prevalence and intensity of hookworm infection are higher in adults compared to children, and several studies showed that worm burdens are the highest in the most advanced age groups of rural communities^6,7^.

Experimental work in mice with GI nematodes such as *Heligmosomoides polygyrus bakeri* proved highly valuable in pinning down the factors required for the initiation, expansion, and regulation of protective immune responses in GI nematode infections. *H. p. bakeri* is a member of the trichostrongylid family which naturally infects the small intestine of house mice^8^. Like several widespread *Trichostrongylus* and *Nematodirus* species infecting the upper GI tract of cattle and small ruminants, *H. p. bakeri* has a strictly enteric life cycle which is initiated by the oral uptake of the third larval stage^9,10^. The L3 rapidly invades the submucosa of the upper small intestine where it develops into the L4. At day 8 p.i, the worms return to the gut lumen, molt into the adult stage, and wrap around the villi in the proximal small intestine^11^. Egg deposition starts at day 10 and *H. p. bakeri* persists for about 4–6 weeks in the most resistant mouse lines, whereas slow responder lines require up to 9 months for complete expulsion^10,12^.

The type 2 cytokines IL-4 and IL-13 are critical for the control of primary and challenge infections with *H. p. bakeri*^12,13^. Recent studies unraveled the line of events driving the type 2 cytokine release from innate lymphoid cells (ILC2) at the very early stages of infection with *H.p. bakeri*. Tuft cells were identified as the source of IL-25, a cytokine driving the release of IL-4, -13 and -5 from ILC2^14^. In addition, ATP released from host cells damaged by the tissue-invasive larval stage was shown to result in adenosine signals eliciting the release of IL-33 which also promotes the activation of ILC2. However, the timely control of primary and challenge infection with *H. p. bakeri* depends on the high number of GATA-3+ CD4+ type 2 T helper cells (Th2) generated in the mesenteric lymph nodes (MLN). Together with follicular T helper cells promoting germinal center formation, Th2 cell-derived IL-4 and IL-13 promote B cell class switching to IgG1 and IgE^15,16^. GATA-3+ Th2 cells rapidly migrate to the infected small intestine and join with the ILC2 population in the attraction of eosinophils, mast cells, and M2 macrophages as well as the support of smooth muscle proliferation and activity. These responses are not fast enough to control the tissue invasive larvae in a primary infection but provide efficient protection against reinfection where most of the larvae are killed by M2 macrophages releasing toxic effector molecules^17^.

In line with parasite control depending on the development of effective Th2 responses, we showed earlier that the differential phenotypical composition of the CD4+GATA-3+ effector T cell population generated by resistant BALB/c compared to susceptible C57BL/6 mice correlated with the outcome of *H. p. bakeri* infection. While both lines generated extensive T effector responses, the GATAT-3+ T cells generated by C57BL/6 mice infected at a young age comprised high proportions of Th2/1 hybrid cells which co-expressed T-bet and IFN-γ together with GATA-3 and modest amounts of Th2 cytokines^18^. In contrast, the response generated by young BALB/c mice was strongly biased for classical Th2 cells, which was associated with the more efficient control of adult worm fecundity compared to age-matched C57BL/6 mice. These differences vanished when BALB/c and C57BL/6 mice were infected a few weeks later in life, with the BALB/c line displaying similar proportions of Th2/1 cells and parasite fecundity as seen in C57BL/6 mice^18﻿^.

Importantly, the differential T helper cell responses translated to a difference in worm fecundity right from the onset of egg production^18^﻿, suggesting that the reproductive fitness of the worms was highly dependent on the immune environment experienced during larval development in the small intestinal tissue. Here, we, therefore, asked if the genotype- and age-dependent resistance to *H. p. bakeri* infection was further linked with the pace of effector cell recruitment to the infected small intestine. Comparing *H. p. bakeri* infected BALB/c and C57BL/6 mice, we found similarly extensive CD4+ T helper cell responses in the gut-draining lymph nodes and congruent kinetics of effector cell release into the blood. However, young BALB/c mice were significantly faster in the recruitment of GATA-3+ T cells to the site of infection compared to age-matched C57BL/6 mice, which was linked with the more robust expression of the small intestinal homing receptor CCR9 binding the chemokine CCL25 which is selectively expressed in the small intestine^19,20^. Accordingly, dendritic cells isolated from mesenteric lymph nodes of infected BALB/c mice displayed higher aldehyde dehydrogenase activity, indicating the production of retinoic acid serving as an inducer of gut-homing receptor expression in T cells^19,21^. However, none of these differences persisted into mature adulthood. Our data therefore show that relatively modest differences in host age can profoundly impact both the phenotype and the migratory activity of T helper cells required for the control of parasite fitness in GI nematode infection.

## Results

### Genetic resistance to *H. p. bakeri* infection is associated with the differential CCR9 expression and Th2 cell recruitment to the infected gut

**Fig. 1:**
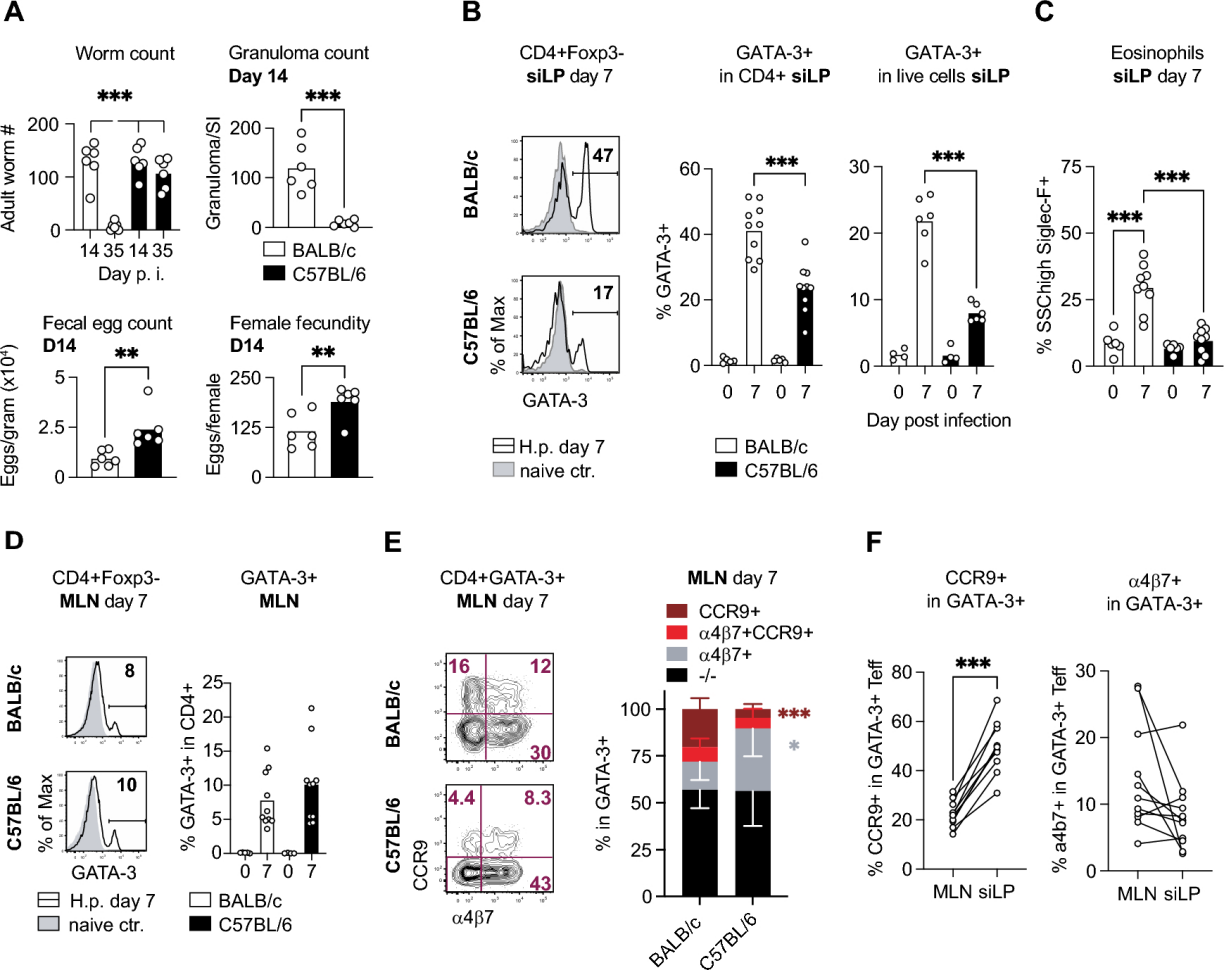
Early gut homing of type 2 effector cells in *H. bakeri* infected BALB/c and C57BL/6 mice. Age and sex-matched BALB/c and C57BL/6 mice were infected with 200 3rd-stage larvae and dissected at day 14 or 35 (A) or 7 (B-G) post-infection next to uninfected controls. (**A**) Top: Adult worm count at day 14 and 35 post infection and granuloma count per small intestine determined on day 14 post-infection. Bottom: nematode eggs per gram feces and eggs produced by individual female worms in culture within 24h post isolation at day 14. Female fecundity is expressed as the mean egg deposition of 8 worms per mouse. Data were pooled from two independent experiments performed with n = 3 mice/group. (**B**) Frequencies of GATA-3+ T effector cells in CD4+Foxp3- T cells from the small intestinal lamina propria (siLP; black line: day 7 p.i.). Cells from naïve controls are shown as filled histograms. Data were pooled from 2-3 independent experiments, each performed with 2–4 infected mice per group. (**C**) Frequencies of SSC high Siglec-F+ eosinophils in siLP cells. Data were pooled as in (B). (**D**) Frequencies of GATA-3+ T effector cells in MLN. Data were pooled as in (B). (**E**) CCR9 and α4β7 expression by GATA-3+ cells isolated from MLN of BALB/c and C57BL/6 mice at day 7 post-infection. Pooled data from 2 independent experiments (each with n = 3/group) are reported as stacked bar graphs. (**F**) Linked CCR9 and α4β7 expression profiles of MLN- and siLP-derived GATA-3+ cells of individual BALB/c mice at day 7 post-infection_._ Data were pooled as in (B). *p ≤ 0.05, **p ≤ 0.01, ***p ≤ 0.001 determined by Kruskal–Wallis test combined with Dunn’s multiple comparison test or Mann–Whitney test.

Comparing BALB/c and C57BL/6 mice on day 14 and 35 post-infection with *H. p. bakeri*, we confirmed the higher resistance of the BALB/c line, evident in the vigorous granulomatous response at the site of larval development, the significantly lower fecal egg counts and in the more rapid expulsion of the adult worms (Fig. 1A). In accordance with earlier work demonstrating the dependence of granuloma formation on type-2 cytokines^12^, we found that cells isolated from the small intestine of BALB/c mice on day 7 post-infection comprised significantly higher frequencies of GATA-3+ Th2 cells, both expressed as a percentage of CD4+ and total live cells, as well as higher frequencies of eosinophils (Fig. 1B, C). However, opposing the clear difference in early mucosal type 2 responses, both mouse lines had similar frequencies and absolute numbers of GATA-3+ cells in the mesenteric lymph nodes draining the site of infection (Fig. 1D and data not shown). To see whether the differential accumulation of Th2 cells in the infected small intestines was associated with differences in the expression of homing receptors targeting lymphocytes to mucosal tissues, we examined MLN-derived GATA-3+ cells for the expression of α4β7, an integrin binding to mucosal addressin cell adhesion molecule-1 (MAdCAM-1) expressed in the microvasculature of the intestine^22^, and CCR9, the receptor for the chemokine CCL25 expressed abundantly in small intestinal tissue^23^. GATA-3+ T cells isolated from MLN on day 7 post-infection comprised high proportions of α4β7+ cells in both mouse lines (Fig. 1E). However, few Th2 cells from C57BL/6 mice expressed the small intestinal homing receptor CCR9 cells as compared to cells from BALB/c mice (Fig. 1E). The linked analyses of GATA-3+ cells derived from MLN and siLP of BALB/c mice on day 7 post-infection showed, expectedly, that CCR9 expression was strongly associated with small intestinal homing of GATA-3+ cells, whereas intestinal Th2 cells were not enriched in α4β7+ cells compared to the MLN derived population (Fig. 1F). Together, these data indicated that the two lines differed in the extent of CCR9 induction in Th2 effector cells, resulting in the delayed accumulation of Th2 cells at the site of infection in susceptible C57BL/6 mice.

### Strong systemic accumulation of GATA-3+ cells in C57BL/6 mice

**Fig. 2:**
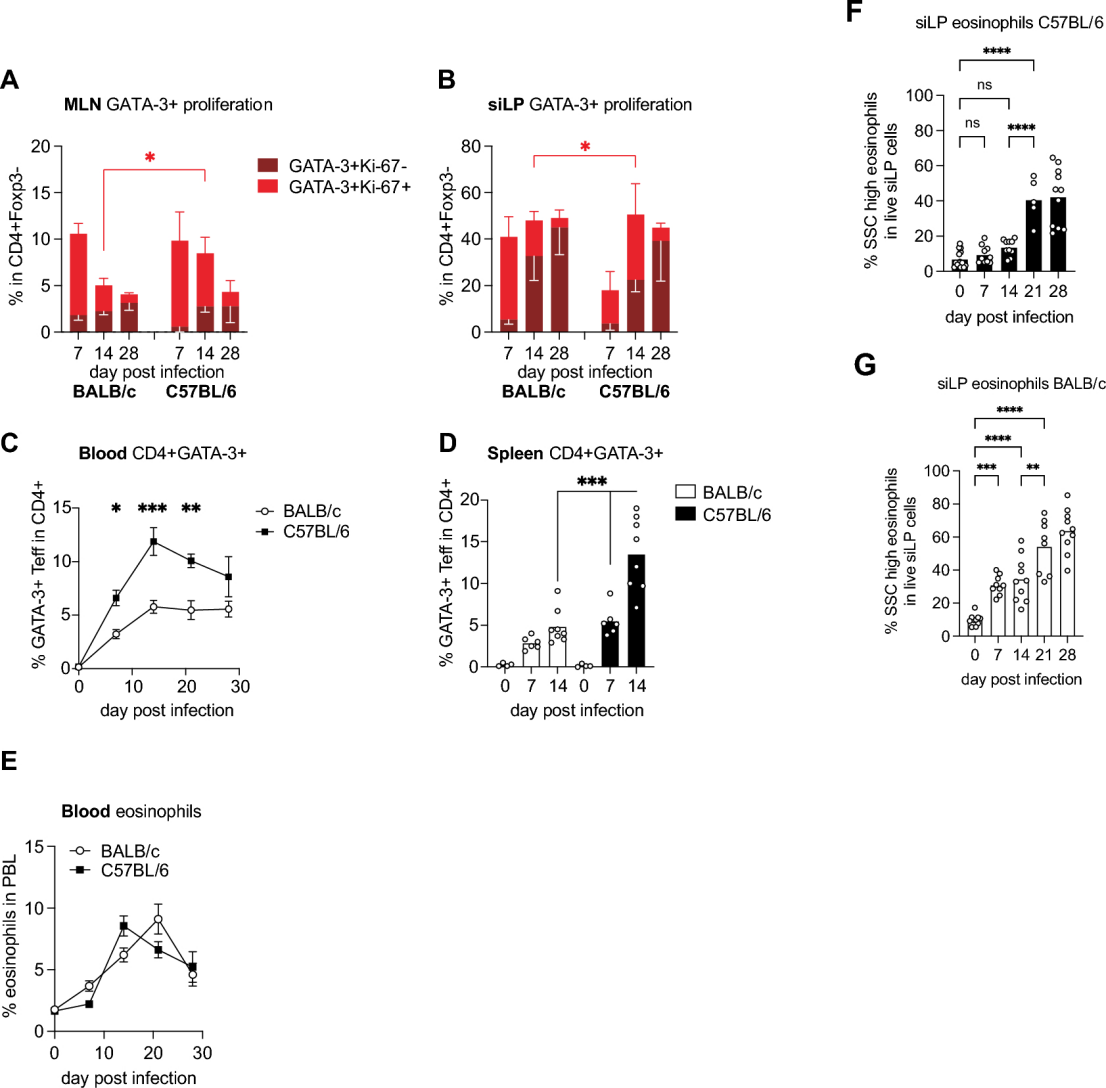
Kinetic of Th2 and innate type-2 response in *H. bakeri* infected BALB/c and C57BL/6 mice. (**A**, **B**) Frequencies of Ki-67+ and Ki-67- cells in CD4+GATA-3+ T cells from MLN (**A**) and siLP (**B**). Data are pooled from 2 to 3 experiments, each performed with 3-4 mice/group and time point. (**C**) Frequencies of GATA-3+ T effector cells in peripheral blood. Data are pooled as described in (A, B). (**D**) Th2 response in spleen. Data are pooled from 2 independent experiments, each performed with 3-5 mice/group. (**E-G**) Eosinophil frequencies in blood (**E**) and siLP cells isolated from C57BL/6 (**F**) and BALB/c mice (**G**) along the course of infection. Data are pooled from 3 independent experiments (d14, 28). Day 21 was analyzed once. *p ≤ 0.05, **p ≤ 0.01, ***p ≤ 0.001 determined by Kruskal–Wallis test combined with Dunn’s multiple comparison test or Mann–Whitney test.

To gain more information on the pace of type 2 response, we investigated the kinetics of GATA-3+ effector cell expansion in both mouse lines. Expectedly, the majority of GATA-3+ cells proliferated in MLN of both mouse lines at day 7 post-infection (Fig. 2A). In BALB/c mice, the frequency of Th2 cells dropped by more than half at day 14 post-infection, associated with the decline in proliferative activity (Fig. 2A). In contrast, Ki-67 expression remained significantly higher in MLN-derived GATA-3+ cells of C57BL/6 mice at day 14, followed by a decline at day 28 to similarly low levels as seen in chronically infected BALB/c mice (Fig. 2A).

A corresponding survey of the small intestinal type-2 response showed that BALB/c mice fully accomplished the accumulation of CD4+GATA-3+ cells in the infected small intestine within 7 days, followed by a similar decline in local Ki-67 expression as seen in MLN (Fig. 2B). This contrasted with C57BL/6 mice which required two weeks to build up maximal small intestinal Th2 responses and still harbored high frequencies of dividing cells in the intestinal GATA-3+ cell pool at day 14 post-infection (Fig. 2B). Matching the prolonged Th2 expansion and limited CCR9 expression in MLN of C57BL/6 mice, GATA-3+ cells accumulated to higher levels in blood and spleen of infected C57BL/6 compared to BALB/c mice along the course of *H. p. bakeri* infection (Fig. 2C, D). We further assessed the proportions of eosinophils in blood and intestine based on Siglec-F expression and side scatter/autofluorescence properties. Fitting the congruent kinetics of Th2 cell induction in MLN, blood eosinophilia developed with similar kinetics in both mouse lines (Fig. 2E). Nevertheless, C57BL/6 mice required about 3 weeks for the significant accumulation of small intestinal eosinophils, whereas the more rapid mucosal accumulation of GATA-3+ cells translated to higher eosinophil frequencies at day 7 and day 14 in the gut of infected BALB/c mice (Figs. 1C and 2F, G). In conclusion, both mouse lines generated strong Th2 responses to the infection with enteric nematodes. However, BALB/c mice accomplished the extensive accumulation of Th2 cells within the time frame required for larval development in the intestinal submucosa. Associated with the limited CCR9 expression of MLN-derived Th2 cells, more susceptible C57BL/6 mice only achieved the full extent of mucosal effector cell accumulation about 1 week later, i.e. after the worms left the tissue and returned to the intestinal lumen.

### Age-dependency of CCR9 expression by Th2 cells

In earlier work, we showed that the higher IFN-γ competence of mature compared to young BALB/c mice translates to the induction of higher numbers of IFN-γ competent Th2/1 hybrid cells in mature mice exposed to *H.p. bakeri* infection. These serve as a parasite-specific source of IFN-γ and thereby counteract the efficient expression of type 2-dependent immune responses, resulting in the lower resistance of mature compared to young mice^18^. Here, we asked whether the decline in resistance along host age was also related to altered kinetics of mucosal effector cell accumulation next to the age-dependent shift from a Th2- to a Th2/1 hybrid biased response.

**Fig. 3:**
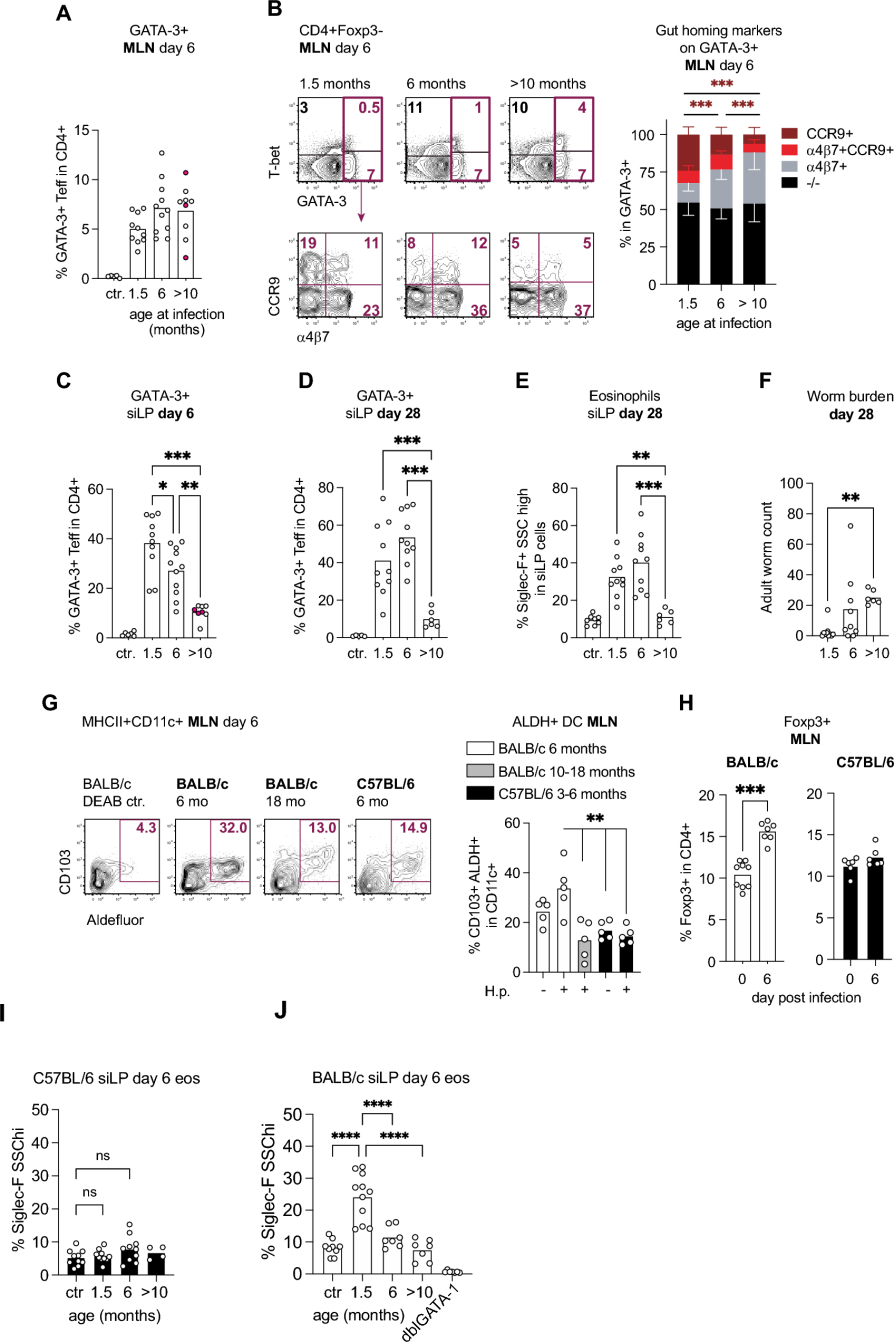
Age-dependent ALDH activity of dendritic cells and CCR9 expression by *H. bakeri*-induced GATA-3+ T cells. BALB/c mice were infected with 200 3rd-stage larvae at the age of 1.5, 6, and more than 10 months and dissected on day 6 (A-C) or 28 post-infection (D, E). (**A**) Frequencies of GATA-3+ cells in MLN. Data are pooled from 2 to 3 experiments each performed with 3–4 mice (age 1.5, 6 months) and 3 experiments with n=3 mice (older than 10 months). Red circles represent the oldest mice which were infected at the age of 18 months. (**B**) Top row: Representative plots of GATA-3 and T-bet expression by CD4+Foxp3- cells derived from MLN on day 6 post-infection. Bottom row: CCR9 and α4β7 expression by GATA-3+ cells. Stacked bars report the profiles of CCR9 and α4β7 expression by GATA-3+ cells of the different age groups. Data were pooled as described in (A). (**C**) Frequencies of GATA-3+ effector cells in siLP at day 6 post-infection. Data were pooled as in (A). Data from mice that were infected at the age of 18 months are marked in red. (**D**) GATA-3+ effector cells in siLP at day 28 post-infection. Data are pooled from 2 experiments, each performed with 3-5 mice/per group (age 1.5 and 6 months) or 2 experiments with n = 3 (older than 10 months). (**E**) Eosinophil frequencies in siLP cells at day 28 post-infection. Data are pooled as described in (D). (**F**) Adult worm count at day 28. Data are pooled as described in (D). (**G**) Representative plots of Aldefluor signals and CD103 expression in MHCII+CD11c+ dendritic cells isolated from MLN at day 6 post-infection. The graph reports the frequencies of CD103+ALDH+ (Aldefluor bright) cells in MHCII+CD11c+ cells derived from MLN of naïve controls and day 6-infected BALB/c and C57BL/6 mice. Data are pooled from 2 independent experiments each performed with 3 mice/group. Some samples were excluded due to insufficient target cells or overtly high proportions of dead cells. (**H**) Frequencies of Foxp3+ Treg in CD4+ cells isolated from MLN of BALB/c and C57BL/6 mice (age 3 months) on day 6 post-infection compared to naïve controls. Data are pooled from 2–3 independent experiments performed with 3–4 mice/group. (**I**) Eosinophil frequencies in siLP cells of C57BL/6 mice at day 6 post-infection. Data are pooled as described in (D). (**J**) Eosinophil frequencies in siLP cells of BALB/c mice at day 6 post-infection. Data are pooled as described in (D). siLP samples from 3 eosinophil-deficient dblGATA-1 mice (day 7 post-infection) were used as gating controls to decipher between eosinophils and intesinal macrophages. *p ≤ 0.05, **p ≤ 0.01, ***p ≤ 0.001, ****p ≤ 0.0001 determined by Kruskal–Wallis test combined with Dunn’s multiple comparison test or Mann–Whitney test.

BALB/c mice infected across an age range of 1.5 to more than 10 months generated similar proportions of GATA-3+ cells in MLN (Fig. 3A). As shown earlier, T-bet+ Th2/1 hybrid cells were more prominent in GATA-3+ cells of mature compared to young mice (Fig. 3B). In addition, we determined a highly significant decrease in CCR9 expression on MLN-derived GATA-3+ cells with age (Fig. 3B). Accordingly, BALB/c mice displayed a gradual delay in the recruitment of GATA-3+ cells to the infected gut along age on day 6 post infection (Fig. 3C). Type 2 T cell recruitment leveled out in 6-month-old mature mice within 4 weeks of infection, whereas mice infected at the age of more than 10 months displayed a long-lasting deficit in small intestinal Th2 cell and eosinophil recruitment (Fig. 3D, E). Consequently, the most advanced age group displayed a significant delay in the completion of adult worm expulsion compared to young mice (Fig. 3F).

Earlier studies showed that the induction of CCR9 depends more strictly on retinoic acid (RA) produced by gut-derived dendritic cells (DC) compared to the upregulation of α4β7 ^21^. We hence assessed the activity of aldehyde dehydrogenase (ALDH), an enzyme required for the conversion of vitamin A to retinoic acid, comparing MLN-derived DC of BALB/c mice infected at the age of 3 to 18 months to those derived from 3 to 6-month-old C57BL/6 mice. Fitting the age-dependent profile of CCR9 expression, CD11c+ DC derived from MLN of young BALB/c mice comprised significantly higher frequencies CD103+ALDH+ DC at day 6 post-infection compared to DC from older individuals of the same line as well as compared to DC from age-matched C57BL/6 mice (Fig. 3G).

As CD103+ migratory DCs are capable of inducing Foxp3+ regulatory T cells^24^, we further assessed the frequencies of Foxp3+ cells in MLN of young BALB/c and C57BL/6 mice at day 6 post-infection compared to uninfected controls. In accordance with the prominent CD103+ALDH+ DC population detected in MLN, BALB/c displayed a significant rise of Treg frequencies at day 6 post-infection, whereas CD4+ T cells of C57BL/6 mice comprised similar proportions of Foxp3+ T cells irrespective of the infection status (Fig. 3H). We further assessed the proportions of eosinophils in the intestines of C57BL/6 and BALB/c at day 6 post-infection and found no rise of tissue eosinophils in any of the infected C57BL/6 age groups (Fig. 3I), whereas young, but not mature BALB/c mice displayed significant tissue eosinophilia early during infection (Fig. 3J). Hence, age- and genotype-dependent differences in the ALDH activity of DC were reflected in the differential CCR9 expression and gut homing kinetics of GATA-3+ effector T cells, as well as in distinct Treg and eosinophil accumulation in MLN and small intestine of nematode-infected mice.

### IFN-γ treatment promotes the systemic accumulation of GATA-3+ cells in BALB/c mice

**Fig. 4:**
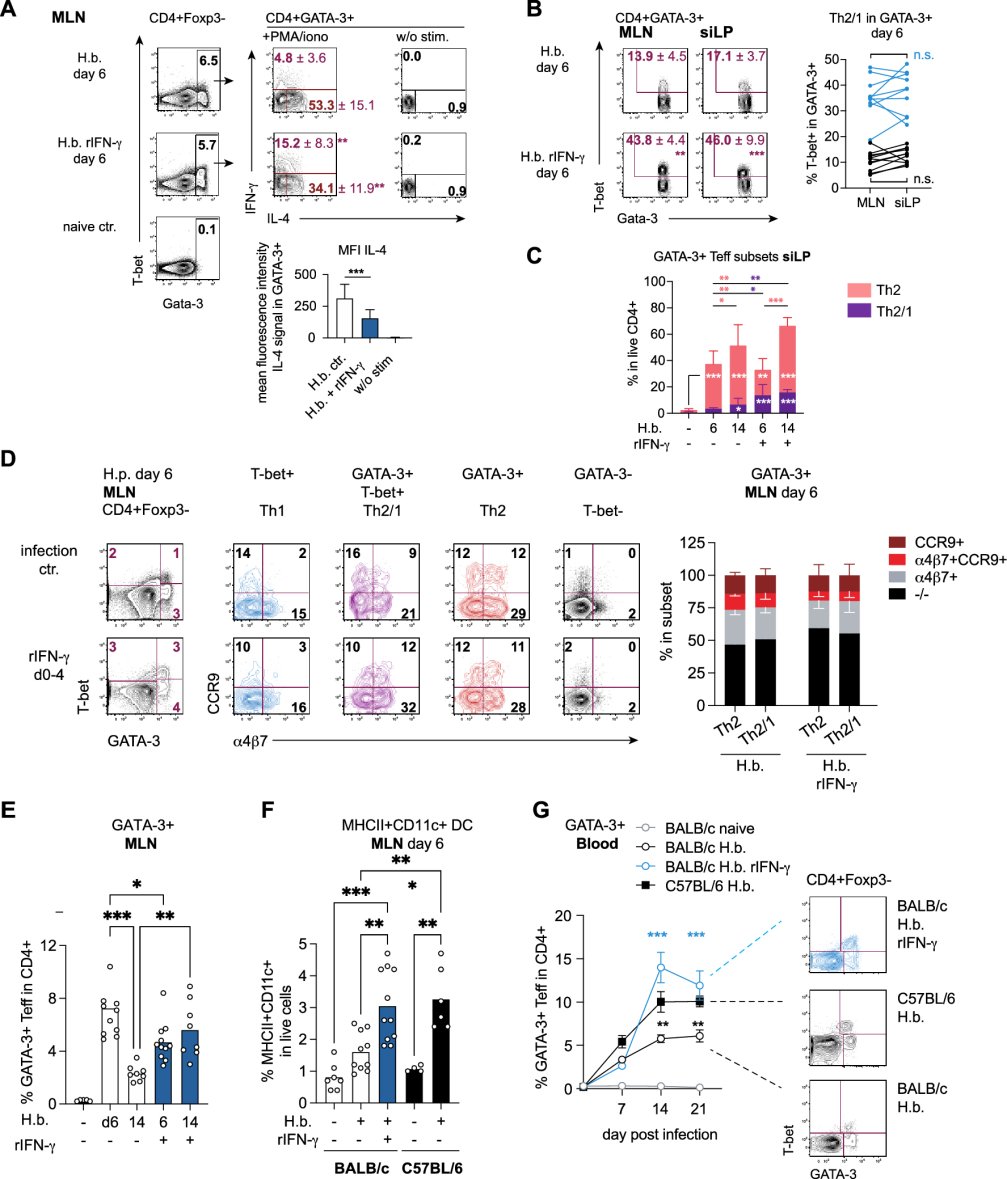
Local and systemic responses following early IFN-γ exposure. BALB/c mice were infected with 200 3rd-stage larvae at the age of 1.5–3 months and treated with IFN-γ (day 0-4) or left untreated. Type-2 responses were analyzed on day 6 or 14 post-infection. (**A**) Top: PMA/ionomycin-stimulated IL-4 and IFN-γ expression by GATA-3+ cells isolated from MLN of untreated infection controls and IFN-γ treated mice on day 6 post-infection. The center and lower plots depict the co-expression of T-bet determined in GATA-3+ cells isolated from MLN and siLP on day 6. Plots report the concatenated data from 5 mice/group. Mean frequencies +/- SD and levels of significance comparing untreated and treated mice are reported in the flow cytometry plots. (**B**) Linked profiles of T-bet co-expression in GATA-3+ cells from MLN and siLP cells of individual mice. Data are pooled from 3 independent experiments, each performed with 3-5 mice/group. (**C**) Frequencies of GATA-3+ T cells in siLP-derived CD4+ T cells at days 6 and 14 post-infection. Day 6 data are pooled from 3 individual experiments, day 14 was analyzed once. (**D**) Left: Representative plots of GATA-3 and T-bet expression by MLN-derived CD4+ cells of an untreated infection control compared to an IFN-γ treated mouse at day 6 post-infection. Colored plots report the expression of CCR9 and α4β7 by Th1, Th2/1 hybrid, and Th2 next to GATA-3-T-bet- cells. Stacked bars report the profiles of CCR9 and α4β7 expression by GATA-3+ cells. Data are pooled from 2 individual experiments performed with 3-4 mice/group. (**E**) Frequencies of GATA-3+ cells in MLN of untreated and IFN-γ-treated mice at day 6 and 14 post-infection. Data are pooled from 3 (day 6) and 2 (day 14) independent experiments. (**F**) Frequencies of MHCII+CD11c+ DC in MLN-derived live cells as determined at day 6 post-infection in untreated versus IFN-γ-treated BALB/c mice. Data from day 6 infected, untreated C57BL/6 mice and the respective naïve controls are included as black bars. BALB/c data were pooled as in D, and C57BL/6 data were derived from 2 individual experiments. (**G**) Kinetic of GATA-3+ T effector cell response determined in peripheral blood. Data are pooled from 3 independent experiments each performed with 4–5 mice/group (day 0–14). Day 21 data derive from one experiment. Colored symbols indicate the level of significance comparing IFN-γ-treated and untreated BALB/c infection controls. Black symbols indicate significance comparing infected BALB/c (untreated) and C57BL/6 mice. Representative FACS plots report the frequencies of GATA-3+ Th2 and GATA-3+T-bet+ Th2/1 hybrid cells, forming the total GATA-3+ T effector pool reported in the graph. *p ≤ 0.05, **p ≤ 0.01, ***p ≤ 0.001 determined by Kruskal–Wallis test combined with Dunn’s multiple comparison test or Mann–Whitney test.

Previously we reported a decline in resistance in BALB/c mice which were driven to the generation of more extensive Th2/1 hybrid responses by IFN-γ treatment during T cell priming^18^. Conversely, C57BL/6 mice turned more resistant when Th2/1 hybrid formation was reduced by blocking of early type 1 signals. Here, we asked whether systemic IFN-γ exposure altered the homing properties of type-2 cells next to shifting the phenotypical composition of the GATA-3+ T cell pool in favor of IFN-γ competent Th2/1 hybrid cells. As expected from our earlier studies, IFN-γ applied to BALB/c mice for the first 5 days of infection resulted in stronger Th2/1 hybrid responses, evident in the more prominent IFN-γ production and diminished IL-4 expression of GATA-3+ T cells isolated from IFN-γ treated mice at day 6 post infection (Fig. 4A). The paired analyses of T-bet co-expression in GATA-3+ cells derived from MLN and siLP of individual mice showed that the cells from both organs comprised similar proportions of Th2/1 hybrid cells in the untreated infection controls and, at a higher level, in mice treated with IFN-γ early during the infection, indicating that naturally as well as experimentally induced Th2/1 hybrid cells migrated to the infected small intestine with similar efficiency as classical Th2 cells (Fig. 4B). Accordingly, the overall frequencies of GATA-3+ effector cells recruited to the gut by day 6 and 14 p.i. were similar between untreated infection controls and IFN-γ treated animals (Fig. 4C). Furthermore, Th2 and Th2/1 cells derived from MLN of untreated infection controls and IFN-γ treated mice displayed similar profiles of CCR9 and α4β7 expression on day 6 post infection. (Fig. 4D). Hence, the exposure to elevated IFN-γ signals strongly promoted the generation of Th2/1 hybrid cells without altering the overall profile of gut homing receptor expression by GATA-3+ effector cells (Fig. 4A, C).

Interestingly, IFN-γ treatment resulted in prolonged type 2 activity in MLN of BALB/c mice, evident in the higher frequencies of GATA-3+ T cells at day 14 p.i. compared to untreated infection controls (Fig. 4E). This effect correlated with the more robust rise of DC frequencies in gut-draining lymph nodes of IFN-γ treated mice early during infection (Fig 4F). IFN-γ-treated BALB/c mice thereby phenocopied the response of untreated C57BL/6 mice in the timely extension of strong type-2 responses localized in MLN (compare Figs. 2A and 4E), in the more robust accumulation of DC in MLN early during infection (Fig. 4F), as well as in the more pronounced systemic accumulation of GATA-3+ T cells comprising high proportions of Th2/1 hybrid cells (Fig. 4G). Hence, elevated IFN-γ signals during T cell priming did not impact the gut homing properties of type 2 cells in BALB/c mice, but rather resulted in more extensive systemic type 2 responses which were similarly enriched in Th2/1 hybrid cells as those found in C57BL/6 mice.

## Discussion

Host genetics have been shown to contribute to the variability in clearing helminth infection, with different mouse strains and sheep breeds displaying varying levels of susceptibility and resistance to helminthiasis^25–28^. BALB/c and C57BL/6 mice display distinct immune profiles, with BALB/c mice exhibiting a bias for Th2-skewed responses, while C57BL/6 mice easily develop Th1 responses characterized by elevated systemic IFN-γ production and enhanced cell-mediated immunity^29,30^. These differences were reported earlier to influence the expression of the gut-homing receptor CCR9, influenced by higher RALDH activity in CD103+ MLN-DCs and an enhanced vitamin A metabolism at steady state in BALB/c compared to C57BL/6 mice^31,32^.

In this work, we show that GATA-3+ cells derived from C57BL/6 and BALB/c mice displayed differential expression of the small intestinal homing receptor CCR9, permitting the faster accumulation of GATA-3+ T cells in the infected small intestine of the resistant BALB/c line. CCR9 specifically supports the homing of T cells to the small intestine due to the selective expression of the chemokine ligand CCL25 in the upper intestinal tract^19,33^. MAdCAM-1, serving as the ligand for the integrin α4β7, is expressed more broadly in mucosal venules along the entire GI tract and therefore expected to result in the more broad dispersal of α4β7+ effector T cells ^34,35^.

The more rapid effector T cell migration to the site of infection in BALB/c mice may allow for the early expression of IL-4Rα-dependent anti-helminth effector molecules typically associated with the protective response in challenge infection with *H. p. bakeri.* In challenged mice, Th2 cells, eosinophils, and M2 macrophages rapidly accumulate around the developing larvae inhibiting their development in an IgG1- and arginase-1 (Arg-1)-dependent manner via L-arginine depletion^36–38^. Accordingly, we show that more rapid accumulation of Th2 cells in primary nematode infection is accompanied by a significant increase of eosinophil frequencies by day 7 in BALB/c mice, whereas a rise in intestinal eosinophils was first seen at day 21 in the small intestine of more susceptible C57BL/6 line. The upper GI tract is enriched in eosinophils at steady state^39^ which involves the action of IL-5 and IL-13 constitutively produced by ILC2 leading to sustained eotaxin expression^40^. The more rapid accumulation of Th2 cells as an additional source of IL-5/13 may therefore join with ILC2 in the faster eosinophil attraction and the support of eosinophil survival in nematode-infected BALB/c mice. It should be noted that the control of parasite fitness in primary infection as well as protection against challenge infection with *H. p*. *bakeri* largely depends on M2 macrophages and not eosinophils^41,17^. We have not assessed differences in M2 marker expression, but an earlier study reported higher expression of arginase-1 among other M2 markers in BALB/c compared to C57BL/6 mice at day 7 post-infection^12^.

Similarly, BALB/c express higher levels of goblet cell-derived RELM-β, an antimicrobial factor that limits the access of luminal *H. p. bakeri* worms to their mucosal food source^42^. Hence, the more rapid accumulation of IL-4/13 competent T cells in the intestine of BALB/c mice may accelerate innate immune response detrimental to larval fitness, and result in higher goblet cell activity impeding the thriving of pre-adult worms as soon as these have returned to gut lumen by day 8 p.i.^11^. Fitting this picture, earlier work showed that the reproductive fitness of the parasite is impaired right from the onset of egg deposition in BALB/c mice, evident in the lower fecal egg counts and individual worm fecundity compared to C57BL/6 mice^12,18^. We also assessed worm fitness by the determination of the ATP content of adult worms and found lower ATP levels in parasites from young BALB/c compared to C57BL/6 mice (data not shown). Recruitment of Th2 cells coupled with the accumulation of eosinophils and M2 in the infected gut enhances granuloma formation along the length of the small intestine and further impacts the fitness of the worms in BALB/c mice^12,43^. In contrast, our data show that the peak of mucosal Th2 responses is seen in ‘slow responder’ C57BL/6 mice only after the worms returned from the tissue niche to the intestinal lumen. Upon relocation, the adult worms are less sensitive to anti-helminth effector mechanisms, such as stress induced by toxic mediators released by alternatively activated M2 macrophages^17^. Furthermore, activated Th2 cells and their cytokines, particularly IL-5 and IL-13, are crucial for eosinophil infiltration and activation^44,45^. In C57BL/6 mice, a delayed initial accumulation of eosinophils in the gut aligns with slower Th2 cell recruitment, a pattern also seen in older BALB/c mice (Fig. 1B & C). However, by days 21–28 of infection, C57BL/6 mice eventually reach comparable levels of Th2 cell accumulation in the gut, similar to BALB/c mice, which is reflected in the corresponding increase in eosinophil numbers (Figs. 2F, G and 3J). Together with the higher proportions of Th2/1 hybrid cells counteracting type 2 responses in C57BL/6 mice in an IFN-γ dependent manner, the limited stress experienced during larval and early adult development due to modest recruitment of adaptive and innate type 2 cells likely benefits parasite fitness at later stages of infection, resulting in the more long-lived infection and higher egg deposition in this mouse line.

The accelerated small intestinal homing of type 2 cells seen in infected BALB/c compared to C57BL/6 mice correlated with the higher CCR9 expression by GATA-3+ cells and the higher ALDH activity of DC isolated from MLN of BALB/c mice early during infection. Retinoic acid (RA), a vitamin A metabolite primarily catalyzed by the enzyme retinaldehyde dehydrogenase 2 (RALDH2) expressed by CD103+ migratory DC in the gut-associated lymphoid tissue, is considered the limiting factor for the imprinting of gut-homing markers during the priming of T cells^46,47^. In accordance with our findings in nematode-infected mice, earlier work reported higher RALDH activity in CD103+ MLN-DCs and an enhanced vitamin A metabolism at steady state in BALB/c compared to C57BL/6 mice^31,32^. Hence, the differential ALDH/RA profile of BALB/c DC compared to DC from aged-matched C57BL/6 mice complies with the distinct patterns of CCR9 expression and, consequently, recruitment of MLN-derived GATA-3+ cells to the small intestine upon infection with enteric nematodes. Furthermore, infected BALB/c mice displayed a significant rise of Treg frequencies in MLN upon infection, which was not seen in C57BL/6 mice. In accordance with the supportive role of RA in Treg induction^48,49^, this finding further indicates the importance of differential ALDH activity in DC for the differences concerning T effector and Treg response between the two nematode-infected mouse lines^50^.

Confirming our previous work investigating mouse age as a distinguishing feature affecting the expression of type 2 responses, the data presented here show that mature mice generate more prominent Th2/1 hybrid responses compared to young BALB/c mice upon *H. p. bakeri* infection^18^. In addition, advanced age affected the mucosal type 2 response via the declining induction of CCR9 in GATA-3+ effector cells (Fig. 3B). Consequently, mature BALB/c displayed a delay in the early recruitment of GATA-3+ T cells to the infected gut, which was compensated for within the following weeks. In contrast, BALB/c mice infected at the age of more than one year displayed a severe and long-lasting deficit in the recruitment of GATA-3+ effectors and eosinophils to the infected gut, resulting in increased worm burden compared to younger BALB/c mice. Of note, type 2 effector cell instruction was not impaired in older mice according to the frequencies of GATA-3+ cells in MLN. Our data rather indicate that a progressive decline in the ability to induce CCR9 expression in CD4+ T cells along age was responsible for the poor support of local type 2 response in older mice. This finding complies with earlier reports on the diminished upregulation of CCR9 by CD4+ T cells of aged mice upon exposure to RA and with the poor RALDH activity reported for MLN-derived DC of aged mice^51,52^. The latter was linked with epigenetic modification of the raldh2 gene promoter region, suggesting that the higher methylation of CpG motifs in MLN DC of aged mice resulted in suppressed RALDH2 expression^52,53^.

In accordance with the strict RA-dependence of CCR9 expression compared to α4β7 induction^54,55^, differential ALDH activity seen in DC of BALB/c and C57BL/6 mice in our study appeared to predominantly affect the expression of CCR9 in CD4+ T cells, whereas the induction of α4β7 integrin was more stable along age, and, in some cases, even enhanced as opposed to the poor CCR9 induction. Furthermore, GATA-3 cells induced in nematode-infected mice consistently comprised three distinctive subsets marked by the single versus co-expression of CCR9/α4β7. However, small intestinal GATA-3+ T cells were enriched in CCR9+, but not in α4β7+ cells when compared to MLN-derived cells, indicating that CCL25-CCR9 interaction prevailed over MAdCAM-1-α4β7 interaction in the recruitment of type 2 T cells to the nematode infected gut. We quantified transcript levels of *ccl25* in the small intestines and found significantly higher infection-driven chemokine expression at day 6 and 14 post-infection(Supplementary Fig. 1). Earlier work reported the levels of *ccl25* expression to decline with age in the small intestines of mice, which may also contribute to the age-related differences in gut accumulation of Th2 cells^56,57^. Notably, though, the small intestinal lamina propria also comprised high frequencies of GATA-3+ CD4+ cells lacking both CCR9 and α4β7, indicating the existence of other means of effector cell recruitment to the small intestine next to CCL25 and MAdCAM-1-mediated T cell attraction. It is also worth mentioning that the increase in worm burden in advanced-aged BALB/c mice was still lower than in C57BL/6 mice, indicating that not all mechanisms of worm clearance are affected alike by increasing host age^58,59^.

In previous work, we demonstrated that Th2/1 cells induced naturally by the infection, or in response to infection under the additional exposure to recombinant IFN-γ, primarily accumulated in the spleen and blood of *H. p. bakeri* infected mice over time^18,60^. We hence investigated whether classical Th2 and Th2/1 hybrid cells differed in the expression of gut-homing receptors. Focusing on the early stage of infection, we show here that both subsets comprise similar proportions of CCR9+ and/or α4β7+ cells. Similar profiles were also seen comparing IFN-γ treated and untreated mice. Accordingly, both types of GATA-3+ cells appeared to home to the infected gut with similar efficiency, evident in the congruent proportions of Th2 and Th2/1 cells comparing the inductive (MLN) and effector site (siLP) on an individual basis at day 6 post-infection. Nevertheless, IFN-γ applied during the onset of nematode infection resulted in the strong accumulation of GATA-3+ effector cells in the blood, confirming our earlier finding of overt systemic effector responses in IFN-γ treated BALB/c mice^18^. Assessing the associated profiles of DC and type-2 responses in MLN, we found higher frequencies of DC in MLN of IFN-γ treated compared to untreated mice early during infection. Similar to DC from infection controls, those were strongly enriched in CD11b+CD103+ DC (data not shown), a subset previously assigned with a unique role in the instruction of Th2 responses^61,62^. The more extensive accumulation of DC was followed by higher frequencies of GATA-3+ T cells in MLN within the 2nd-week p.i., alongside the pronounced accumulation of GATA-3+ cells in circulation. These consequences of IFN-γ exposure mirrored the responses of highly IFN-γ competent C57BL/6 mice which harbored more T-bet+IFN-γ + Th1 cells in the spleen, MLN, and small intestines compared to BALB/c mice (see reference^18^) and therefore not only produced higher proportions of Th2/1 hybrid cells but also displayed a stronger accumulation of peripheral GATA-3+ T effector cells in blood than BALB/c mice (Fig. 4G and reference^18^). This suggests that elevated IFN-γ signaling early during infection can actually promote type 2 effector cell responses. Of note, an earlier study showed that small amounts of IFN-γ are required for optimal in vivo priming of IL-4+ T cells and the antigen-driven expansion of IL-4+ cells^18^. However, in our system, the IFN-γ driven bias in favor of Th2/1 hybrid generation resulted in early recruitment of Th2/1 hybrid cells with limited IL-4 competence to the site of infection, followed by the strong systemic accumulation of a hybrid biased effector population in IFN-γ treated BALB/c mice. This contrasts with the Th2-biased response and rapid recruitment of effector cells to the infected gut of unmanipulated young BALB/c mice, allowing for the more efficient control of nematode fitness early during infection.

In summary, our data show that the high resistance of BALB/c compared to C57BL/6 mice against enteric nematode infection correlates with the efficient induction of CCR9 expression in GATA-3+ effector cells and the rapid accomplishment of effector cell recruitment to the infected gut. While the instruction of GATA-3+ effector T cells was similar between resistant BALB/c and susceptible C57BL/6 mice as well as across a wide age range of BALB/c mice investigated in the current study, the more limited ALDH activity of DC and CCR9 induction in GATA-3+ cells translated to delayed effector responses in the infected gut of older mice. The homing properties of type 2 cells induced in young mice remained undisturbed under experimental IFN-γ exposure. However, a bias for the instruction of Th2/1 hybrid responses, paired with the limited gut homing properties of type-2 cells, likely contributes to the declining resistance along age as well as to the delayed control of *H. p. bakeri* infection in genetically susceptible mice.

## Material and methods

### Mice, infections, and treatment

All animal experiments were performed in accordance with the National Animal Protection Guidelines and approved by the German Animal Ethics Committee for the Protection of Animals (G0176/17, G0113/15, H0438/17, G0207/19). Female BALB/c and C57BL/6 mice were from Janvier (Saint-Berthevin, France). Mice were infected with 200 3rd-stage larvae of *H. p. bakeri* by oral gavage. In some experiments, BALB/c mice were treated with recombinant IFN-γ (2.5 µg in 200 µl PBS) given intraperitoneally twice daily for the first 5 days of infection. All mice used in these experiments were anesthetized with xylazine and ketamine and sacrificed by cervical dislocation.

### Parasitological parameters

Fecal egg counts were determined for weighed pellets collected from individual mice. The material was mixed with water (1 ml) followed by the addition of saturated sodium chloride solution (6 ml). After mixing, egg counts were determined using McMaster chambers. The fecundity of individual female worms was determined by counting the eggs released after 24h of cultivation on 96 well plates in serum-free RPMI supplemented with 200U/ml penicillin and 200mg/ml streptomycin (PAA, Wien, Austria).

### Cell isolation

Blood samples were collected into FACS buffer (PBS; PAN-Biotech with 0.2% BSA, 2 mM EDTA). MLN and spleen were processed into single cell suspensions using 70µl cell strainers (BD Biosciences, San Jose, CA, USA). After washing in RPMI (1% FCS, 100U/ml penicillin, 100 mg/ml streptomycin (PAA, Wien, Austria), red blood cells were lysed in splenocyte samples, followed by two further washing steps (eBioscience 1x RBC Lysepuffer). Cells from the small intestinal lamina propria were isolated as described earlier^16^.

### Flow cytometry

For the detection of gut homing markers, cells isolated from lymphatic organs, blood, and small intestinal tissue were stained for CCR9 (clone CW-1.2; PE-Cy7) and α4β7 (clone DATK32, biotin) at 37 °C for 30 min, followed by incubation with a fixable viability dye (eFluor506 or eFluor780) on ice for 5 min. Erythrocytes were removed from blood samples using the BD FACS lysis solution. After labeling CD4 (clone RM4-5; Alexa 700, Brilliant Violet 510, or PerCP), cells were fixed using fix/perm buffer (Thermo Fisher) for 30 min at room temperature and stained intracellularly using the following reagents: FoxP3 (clone FJK-16s; PE-eFluor610, Alexa 488, or PerCP-Cy5.5), GATA-3 (clone TWAJ; eFluor 660), T-bet (clone 4B10; PE), and Ki-67 (SolA15, eFluor 450 or PE-Cy7). Streptavidin coupled to Brilliant Violet 605 was used as a secondary conjugate. In some experiments, an antibody against Siglec-F (clone: E50-2440; PerCP-Cy5.5) was included in surface stains. Dendritic cells were analyzed using antibodies against MHCII (clone M5/114.15.2; APC), CD11c (clone N418; Brilliant Violet 421), and CD103 (clone 2E7, APC). Aldehyde dehydrogenase (ALDH) activity was determined using the Aldefluor^TM^ kit (Stemcell Technologies). Non-specific binding of antibodies was prevented by adding FcγRII/III blocking antibody (clone 93). All antibodies and other reagents were from BioLegend, Thermo Fisher, or BD Biosciences. Cells were acquired on FACSCantoTM II (BD Biosciences) or FACSAriaTM III (BD Biosciences) and data was analyzed using FlowJo (Tree Star Inc., Ashland, USA).

### Gene expression analysis

Frozen small intestinal samples were prepared for RT-qPCR analysis according to the manufacturer’s protocol and using the InnuPrep RNA Mini Kit (Analytik Jena, Jena, Germany). Total RNA was extracted from the samples and mRNA was transcribed to cDNA using the High-Capacity RNA-to-cDNA kit from Applied Biosystem (ThermoFisher; Waltham, MA, USA). Amplifications were performed in duplicate in a final volume of 20 μL containing 10 μL of 2X SYBR Green I Master Mix and 5 μM of CCL25 primer (Table S1). Gene expression of CCL25 was normalized with GAPDH housekeeper and relative expression was calculated using the 2-ΔΔCT method^63^. Data is expressed as the mean and standard error of the mean (SEM).

### Statistics

All statistical analyses were performed using GraphPad Prism Software (San Diego, CA, USA). Normality was tested with the Shapiro–Wilk test, followed by ordinary one-way-ANOVA or Kruskal–Wallis test and Tukey’s or Dunn’s multiple comparison test. Comparisons of the two groups were performed with an unpaired t-test or Mann–Whitney test.

## Supplementary Information

Supplementary Information 1.

Supplementary Information 2.

## Data Availability

The datasets used and/or analysed in support of the current study are available from the corresponding author upon reasonable request.
