# Supplementary material for: Differential resistance to nematode infection is associated with the genotype- and age-dependent pace of intestinal T cell homing
Source: Sci Rep. 2025 Feb 5;15:4424. doi: 10.1038/s41598-024-76204-4 (PMC11799532; doi:10.1038/s41598-024-76204-4)
Supplement: Supplementary file 1 — Supplementary Information 1. [file 41598_2024_76204_MOESM1_ESM.pdf]

Supplementary Figure 1:

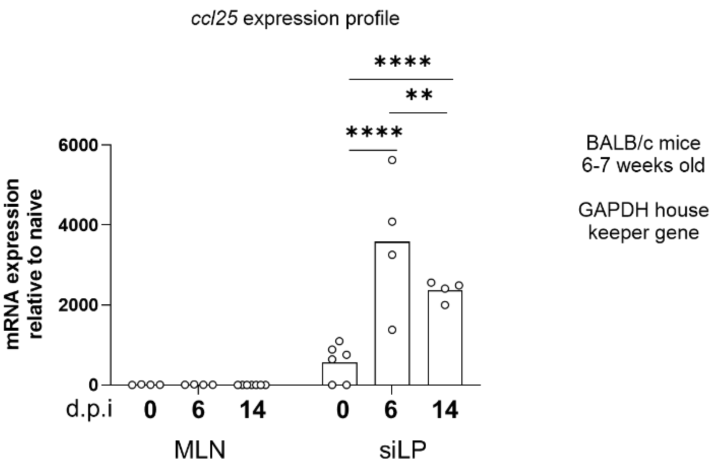

Supplementary Fig. 1. *ccl25* gene expression profiles in MLN and duodenal tissue quantitative real time PCR

Bar graph representing mean relative expression of mRNA (±SEM) of *ccl25* in MLN and small intestinal tissue (duodenum). Data were tested for normality and statistical significance was determined by one-way ANOVA followed by Tukey's multiple comparisons test, $**p \leq 0.01$, $****p \leq 0.0001$.
