# Supplementary material for: Differential resistance to nematode infection is associated with the genotype- and age-dependent pace of intestinal T cell homing
Source: Sci Rep. 2025 Feb 5;15:4424. doi: 10.1038/s41598-024-76204-4 (PMC11799532; doi:10.1038/s41598-024-76204-4)
Supplement: Supplementary file 2 — Supplementary Information 2. [file 41598_2024_76204_MOESM2_ESM.pdf]

Supplementary Table 1:

Table 1: primer sequence for ccl25 gene used for the quantitative real time PCR

Primer name	Forward sequence	Reverse sequence	Company
Mouse ccl25	5‘-AAGGCTAGTCCACTGGAAGAGC-3‘	5‘-GTGGCACTCCTCACGCTTGTAC-3‘	Tib Molbiol
